# High-sensitivity versus conventional troponin in the emergency department for the diagnosis of acute myocardial infarction

**DOI:** 10.1186/cc10270

**Published:** 2011-06-10

**Authors:** Yonathan Freund, Camille Chenevier-Gobeaux, Pascale Bonnet, Yann-Erick Claessens, Jean-Christophe Allo, Benoit Doumenc, François Leumani, Claudine Cosson, Bruno Riou, Patrick Ray

**Affiliations:** 1 Department of Emergency Medicine and Surgery, Hôpital Pitié-Salpétrière, Assistance Publique-Hôpitaux de Paris (APHP), Université Pierre et Marie Curie-Paris 6 (UPMC), 47-83 boulevard de l'hôpital, F-75651 Paris cedex 13, France; 2 Department of Biochemistry, Hôpital Cochin-Hôtel Dieu, APHP, 27 rue du Faubourg Saint-Jacques, F-75679 Paris cedex 14, France; 3 Department of Emergency Medicine, Hôpital Cochin-Hôtel Dieu, APHP, Université Paris Descartes-Paris 5, 27 rue du Faubourg Saint-Jacques, F-75679 Paris cedex 14, France; 4 Department of Emergency, Hôpital Bichat, APHP, 46 rue Henri Huchard, F-75018, Paris, France; 5 Department of Biochemistry, Hôpital Bicêtre, APHP, 78 rue du Général Leclerc 94270, Le Kremlin-Bicêtre, France; 6 INSERM UMRS 956, UPMC, 91 Boulevard de l'Hôpital, F-75013 Paris, France

## Abstract

**Introduction:**

Recently, newer assays for cardiac troponin (cTn) have been developed which are able to detect changes in concentration of the biomarker at or below the 99th percentile for a normal population. The objective of this study was to compare the diagnostic performance of a new high-sensitivity troponin T (HsTnT) assay to that of conventional cTnI for the diagnosis of acute myocardial infarction (AMI) according to pretest probability (PTP).

**Methods:**

In consecutive patients who presented to our emergency departments with chest pain suggestive of AMI, levels of HsTnT were measured at presentation, blinded to the emergency physicians, who were asked to estimate the empirical PTP of AMI. The discharge diagnosis was adjudicated by two independent experts on the basis of all available data.

**Results:**

A total of 317 patients were included, comprising 149 (47%) who were considered to have low PTP, 109 (34%) who were considered to have moderate PTP and 59 (19%) who were considered to have high PTP. AMI was confirmed in 45 patients (14%), 22 (9%) of whom were considered to have low to moderate PTP and 23 (39%) of whom were considered to have high PTP (*P *< 0.001). In the low to moderate PTP group, HsTnT levels ≥ 0.014 μg/L identified AMI with a higher sensitivity than cTnI (91%, 95% confidence interval (95% CI) 79 to 100, vs. 77% (95% CI 60 to 95); *P *= 0.001), but the negative predictive value was not different (99% (95% CI 98 to 100) vs. 98% (95% CI 96 to 100)). There was no difference in area under the receiver operating characteristic (ROC) curve between HsTnT and cTnI (0.93 (95% CI 0.90 to 0.98) vs. 0.94 (95% CI 0.88 to 0.97), respectively).

**Conclusions:**

In patients with low to moderate PTP of AMI, HsTnT is slightly more useful than cTnI. Our results confirm that the use of HsTnT has a higher sensitivity than conventional cTnI.

## Introduction

Early detection of acute myocardial infarction (AMI) remains a major concern, with approximately 15 million patients per year presenting to US emergency departments (EDs) with symptoms suggestive of the diagnosis [1,2]. Among such patients, a strong association between elevated cardiac troponin (cTn) levels and myocardial necrosis has been clearly demonstrated [3-5]. Conventional cTn has revolutionised the management of patients presenting with suspected acute coronary syndrome (ACS), including risk stratification of ACS, and the use of cTn measurements is recommended by current guidelines [6]. A cutoff point at the 99th percentile has been endorsed, as values above this level have repeatedly proven to be associated with adverse cardiovascular outcomes, including death [7-13]. However, the delay (4 to 6 hours, and 12 hours for peak level) in its elevation remains of concern, since it can delay AMI diagnosis and its treatment and increases the burden on EDs. Thus, cTn measurement does not reliably exclude AMI without repeated negative measurements over the course of 4 to 6 hours. These last years, newer assays have been developed, and High Sensitivity Troponin (HsTn) has been associated with higher sensitivity and NPV than conventional cTn. Recent studies have shown excellent diagnostic performance, even with early presentation to the ED [14], and a better diagnostic accuracy than cTn [15]. However, the latter studies did not evaluate the diagnostic accuracy of high-sensitivity troponin T (HsTnT) according to the pretest probability (PTP) of AMI. For example, ST elevation on an electrocardiogram of a patient with chest pain would be diagnosed as AMI, and then the patient would undergo cardiac catheterization without any measurement of a cardiac biomarker. Furthermore, one of the potential strengths of HsTnT might be the exclusion of AMI earlier than it would be with conventional cTn measurement as suggested by previous studies [15]. Therefore, the objectives of the current study were to confirm whether HsTnT is more sensitive than conventional cTnI to detect AMI according to the patient's PTP.

## Materials and methods

### Clinical setting

During the period from August 2005 to January 2007 in three urban teaching hospitals, we prospectively enrolled consecutive hospital outpatients (> 18 years of age) who presented to the ED with chest pain suggestive of ACS with the onset or peak occurring within the previous 6 hours. Patients with acute or chronic kidney failure requiring dialysis were excluded. The study was performed according to the principles of the Declaration of Helsinki and approved by the local ethics committee (Comité de Protection des Personnes Ile-de-France VI, CHU Pitié-Salpétrière Hospital, Paris, France). Because routine medical care was unchanged, waiver of informed consent was authorised. We followed most of the recommendations concerning the reporting of diagnostic studies set forth by the Standards for Reporting of Diagnostic Accuracy initiative [16].

### Routine assessment

As part of the routine assessment in our institutions, all patients underwent an initial clinical evaluation that included clinical history, a physical examination, 12-lead electrocardiography (ECG), pulse oximetry, routine blood tests and chest X-rays. After these routine tests were done, and before cardiac biomarker results were available, ED physicians were asked to offer an 'empirical' clinical probability of AMI (low, medium or high PTP) based on cardiovascular risk factors, type of chest pain, physical findings and electrocardiogram abnormalities [17,18]. Conventional cardiac troponin I (cTnI) was measured at presentation and, if needed, was repeated after 3 to 9 hours as long as it was clinically indicated. Thus, according to the diagnosis of non-ST elevation MI (NSTEMI) or ST elevation MI (STEMI), the patients were admitted either to the cardiology unit for further evaluation and treatment or directly to the catheterization laboratory for primary percutaneous coronary intervention. However, the timing and treatment of patients were left to the discretion of the attending physicians according to the suspected diagnosis. ED physicians in charge were blinded to the results of HsTnT, and biologists were blinded to the emergency diagnosis suspected by physicians.

To determine the etiologic diagnosis of chest pain at presentation for each patient, two independent experts (ED physicians) who were blinded to the results of HsTnT reviewed all available medical records (including patient history, physical findings, results of laboratory and radiologic testing, ECG, echocardiography, cardiac exercise test, coronary angiography and summary chart at discharge) pertaining to the patient from the time of ED presentation to 30-day follow-up. In the event of diagnostic disagreement, cases were reviewed and adjudicated in conjunction with a third expert (also an ED physician).

AMI was diagnosed according to the joint European Society of Cardiology/American College of Cardiology/American Heart Association/World Heart Federation Task Force redefinition of MI guidelines [6]. Diagnosis of AMI required a cTnI increase above the 10% coefficient of variation (CV) value associated with at least one of the following: symptoms of ischaemia, new ST-T changes or a new Q wave on an electrocardiogram, imaging of new loss of viable myocardium or normal cTnI on admission. Unstable angina was diagnosed in patients with constant normal cTnI levels and a history or clinical symptoms consistent with ACS. Predefined further diagnostic categories included AMI (STEMI with the presence of ST-segment elevation in at least two continuous leads on ECG, new onset of left bundle branch block or NSTEMI), unstable angina, and a third group including cardiac but not coronary symptoms (for example, stable angina, myocarditis, arrhythmias and heart failure), noncardiac symptoms (for example, pulmonary embolism) and chest pain of unknown origin.

To assess the influence of renal function on cTn measurement accuracy, the creatinine level was measured in each patient and then renal function was estimated using the Modification of Diet in Renal Disease study equation [19].

### Biochemical analysis

In two EDs (Cochin Hospital and La Pitié Salpêtrière Hospital, Paris, France), plasmatic cTnI concentrations were routinely measured on an Xpand HM analyzer using the Cardiac Troponin I one-step enzyme immunoassay system (Siemens Healthcare Diagnostics Inc., Newark, NJ, USA). The measurement range extended from 0.04 to 40.00 μg/L. The threshold for this method (0.14 μg/L) corresponds to the lowest substrate concentration that can be reproducibly measured with a CV ≤ 10%. In the remaining ED (Bicêtre Hospital, Le Kremlin-Bicêtre, France), plasmatic cTnI concentrations were routinely measured on an Access analyser (Beckman Coulter, Inc., Brea, CA, USA). The measurement range of this one-step chemiluminescence immunoassay extends from 0.01 to 100.00 μg/L. The threshold (10% CV) given by the manufacturer is 0.06 μg/L.

### HScTnT measurement

Heparinised samples collected upon admission and, if available, samples collected 3 to 9 hours later were analysed. Plasmatic highly sensitive cardiac TnT (HScTnT) concentrations were measured using the HScTnT one-step electrochemiluminescence immunoassay on an Elecsys 2010 analyzer (Roche Diagnostics, Meylan, France). The measuring range extended from 0.003 to 10 μg/L. The threshold for this method is 0.014 μg/L and corresponds to the 99th percentile. The CV was found to be < 10% at 0.014 μg/L. In our laboratory, CVs obtained in Roche Diagnostics quality controls containing 0.027 and 2.360 μg/L of HScTnT were < 4%. These analytical performance levels were in accordance with data provided by the manufacturer.

### Statistical analysis

Continuous variables are presented as means ± SD or medians (25th to 75th percentile), and categorical variables are expressed as numbers and percentages. Continuous variables were compared by using the Mann-Whitney *U *test, and categorical variables were assessed using Pearson's χ^2 ^test. Correlations among continuous variables were assessed using the Spearman's rank correlation coefficient. Receiver operating characteristic (ROC) curves were constructed to assess the sensitivity and specificity, positive predictive value (PPV) and negative predictive value (NPV), positive likelihood ratio (LR^+^) and negative likelihood ratio (LR^-^) (all data presented with their 95% confidence intervals (95% CIs)) throughout the concentrations of cTnI and HScTnT to compare the accuracy of these markers in the diagnosis of AMI. Comparison of areas under the ROC curve was performed [20]. As this comparison is recognised as potentially insensitive, the net reclassification index (NRI) method was used as recently described [21]. For tests with binary outcomes (such as cTn for the diagnosis of AMI), NRI is defined as the gain in certainty of the first test (cTnI) minus the gain in certainty of the second test (HScTnT) or, alternatively stated, the difference of the sum of the sensitivity and specificity expressed as follows:

NRI is the combination of four components: the proportion of individuals with events who move up or down in a category and the proportion of individuals with nonevents who move up or down in a category. Table 1 is a contingency table comparing diagnostic classifications according to cTnI and HsTnT, with shifts between the two classifications, to represent the possible benefit of HScTnT in terms of the number of patients correctly reclassified. As stated in the Routine assessment subsection above, we separated the study population into two groups: one included the patients assessed as having low or moderate PTP of AMI and the other assessed as having high PTP of AMI.

**Table 1:** Contingency data according to pretest probability^a^

	All patients		
Patient characteristics	AMI	No AMI	Total
Positive cTnI	32	9	41
Negative cTnI	13	263	276
Total	45	272	317
Positive HsTnT	42	48	90
Negative HsTnT	3	224	227
Total	45	272	317
	**Low to moderate PTP**		
	**AMI**	**No AMI**	**Total**
Positive cTnI	17	7	24
Negative cTnI	5	229	234
Total	22	236	258
Positive HsTnT	20	36	56
Negative HsTnT	2	200	202
Total	22	236	258

^a^Net reclassification improvement (NRI) from the use of highly sensitive troponin T (HsTnT) was 7.9% (95% CI = 0.9 to 14.9; *P *= 0.034). Comparison of the model including HsTnT with cTnI was significant for low PTP patients (NRI = 10.3%, 95% CI = 1.9 to 18.7; *P *= 0.027), but NRI was not significantly different in moderate PTP patients (NRI = 11.6%, 95% CI = -0.5 to 23.7; *P *= 0.084) or in high PTP patients (NRI = -14.4%, 95% CI = -32.6 to -3.6; *P *= 0.181).

All hypothesis testing was two-tailed, and *P *< 0.05 was considered statistically significant. Statistical analysis was performed using StatView for Windows version 5.0 software (SAS Institute, Cary, NC, USA) and MedCalc software for ROC analysis (MedCalc Software, Mariarkerke, Belgium). Graphs were built with GraphPad Prism 5 software (GraphPad Software Inc., La Jolla, CA, USA).

## Results

After 18 months, 317 consecutive patients were enrolled in the study. The baseline characteristics of the patients are shown in Table 2. The mean age of the patients was 57 ± 17 years (range, 40 to 90 years), and 205 (65%) were men. There were significant proportions of older adult patients (31% patients were age 65 years or older, *n *= 98) and patients with a history of cardiovascular events (26%, *n *= 83). Chest pain was considered typical of ACS in 43% (*n *= 136) of the patients. In our study population, 149 patients (47%) were assessed as having a low PTP of AMI, 109 patients (34%) were assessed as moderate and 59 patients (19%) were assessed as high. AMI was confirmed in 45 patients (14%), 13 of whom had sustained STEMI, and all of these 13 patients were in the high PTP group; 32 of the patients had sustained NSTEMI. Table 2 shows that patients in the two groups (high PTP and low or moderate PTP) had significantly different characteristics. There was a higher rate of a personal history of AMI in the high PTP group and a higher final diagnosis of AMI (39% vs. 9%) in the high PTP group (*P *< 0.001). At 30 days after admission, there were three deaths (two in the AMI group and one in the other cause group) and four relapses of ACS (all in the AMI group).

**Table 2:** Baseline characteristics of the population according to the pretest probability^a^

Population characteristics	All patients	Low or moderate PTP	High PTP	*P *value*
Number of patients	317	258	59	
Age, years	57 ± 17	56 ± 17	60 ± 17	0.168
Men	205 (65)	166 (64)	39 (66)	0.88
Systolic BP, mmHg	141 ± 28	141 ± 27	144 ± 30	0.396
Diastolic BP, mmHg	80 ± 16	80 ± 16	82 ± 16	0.428
Heart rate, beats/minute	85 ± 45	84 ± 23	80 ± 19	0.177
Pulse oxymetry, %	97 ± 3	97 ± 3	97 ± 2	0.651
TIMI risk score	1 (0 to 3)	1 (0 to 2)	2 (1 to 4)	< 0.001
Family history of CAD	100 (32)	77 (30)	23 (59)	0.161
Personal history of CAD	83 (26)	56 (22)	27 (46)	0.0003
Dyslipidemia	113 (36)	86 (33)	27 (46)	0.069
Smoking	128 (40)	99 (38)	29 (49)	0.145
Diabetes	44 (14)	31 (12)	13 (22)	0.059
Hypertension	116 (37)	89 (34)	27 (46)	0.134
History of heart failure	21 (7)	14 (5)	7 (12)	0.083
Typical thoracic pain	136 (43)	105 (41)	31 (53)	0.11
Positive cTnI at admission	41 (13)	24 (9)	17 (29)	< 0.001**
eGFR, mL/minute/1.73 m^2^	77 (62 to 94)	77 (64 to 94)	76 (56 to 91)	0.187
				
Treatment within first 24 hours after admission				
Aspirin	119 (38)	79 (31)	40 (68)	<0.001
Clopidogrel	54 (17)	29 (11)	25 (42)	< 0.001
LMWH	68 (21)	41 (16)	27 (46)	< 0.001
Anti GPIIb/IIIa	3 (1)	1 (0)	2 (3)	0.09
Coronarography	83 (26)	51 (20)	32 (54)	< 0.001
				
Outcomes				
Hospital admission	192 (61)	140 (54)	52 (88)	< 0.001
Admission to CCU	134 (42)	88 (34)	46 (78)	< 0.001
				
Final diagnosis				
AMI	45 (14)	22 (9)	23 (39)	< 0.001
STEMI	13 (4)	0 (0)	13 (22)	< 0.001
NSTEMI	32 (10)	22 (9)	10 (17)	< 0.001
Unstable angina	11 (3)	4 (2)	7 (12)	< 0.001
Other diagnosis	261 (82)	232 (90)	29 (49)	< 0.001***

^a^AMI, acute myocardial infarction; BP, blood pressure; CAD, coronary artery disease; cTnI, conventional troponin I; eGFR, estimated glomerular filtration rate; LMWH, low-molecular-weight heparin; anti-GPIIb/IIIa, Anti-glycoprotein IIb-IIIa; CCU, cardiologic care unit; NSTEMI, non-ST elevation myocardial infarction; PTP, pretest probability; STEMI, ST elevated myocardial infarction. TIMI, Thrombolysis in Myocardial Infarction. Results are expressed as means ± standard deviations, medians (25th to 75th percentile) or *n *(%); *statistical comparisons are between low to moderate PTP and high PTP groups unless otherwise indicated; **P > 0.14 μg/L in Pitie-Salpetriere and Cochin, *P *> 0.06 μg/L in Bicêtre; ***Statistical comparison including stable angina (*n *= 63), pulmonary embolism (*n *= 16), myopericarditis (*n *= 43), heart failure (*n *= 5) and others.

### HsTnT diagnostic performances

**Figure 1:**
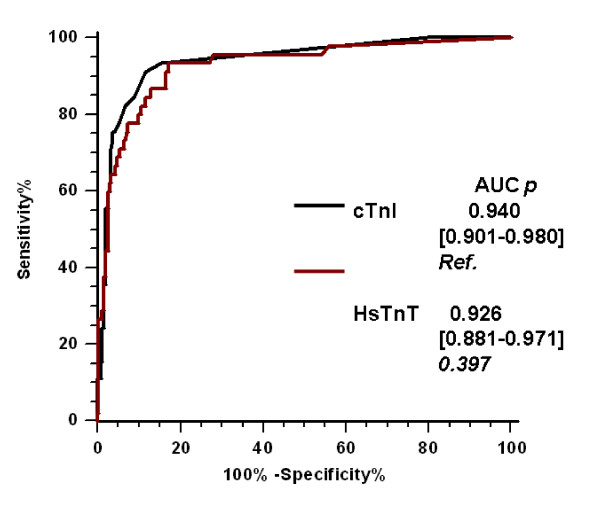
**ROC curves for the diagnosis of AMI**. Values were log-transformed before analysis. AUC: area under the curve; cTnI: conventional troponin I; HSTnT: highly sensitive troponin T.

The area under the ROC curve (AUC) for the diagnosis of AMI was 0.940 (95% Confidence Intervall 0.901 to 0.980) (*P *< 0.001) for initial cTnI compared to 0.926 (0.881 to 0.971) (*P *< 0.001) for HsTnT. However, there was no significant difference between AUCs (Figure 1). ROC analysis indicated an optimal threshold of HsTnT for the diagnosis of AMI at 0.014 μg/L, with a high sensitivity of 89% (78 to 98) and a high specificity of 82% (78 to 87). The overall diagnostic accuracy of HsTnT was not significantly different compared to that of cTnI, regardless of PTP. Similar results (data not shown) were observed when we considered only NSTEMI patients (that is, after exclusion of the 13 STEMI patients). For the diagnosis of AMI, the sensitivities of HsTnT were higher and the specificities were lower than those of cTnI, regardless of PTP (Table 3). When we assessed the low and moderate PTP populations, the sensitivity of HsTnT was higher (91% (79 to 100) vs. 77% (60 to 95)) but NPV was not (99% (96 to 100) vs. 98% (95 to 99) for cTnI).

**Table 3:** Diagnostic accuracy of HScTnT compared to that of cTnI for the diagnosis of AMI according to pretest probability^a^

Patient characteristics	Sensitivity	Specificity	PPV	NPV	Acc	LR^+^	LR^-^
All patients (*N *= 317)							
Positive cTnI	71 (55-84)	97 (94 to 98)	78 (62 to 89)	95 (92 to 97)	93 (90 to 96)	21.5 (20.1 to 22.9)	0.32 (0.23 to 0.36)
Positive HScTnT	93 (89 to 100)*	82 (77 to 87)*	47 (36 to 58)*	99 (96 to 100)	84 (79 to 88)*	5.3 (4.8 to 5.8)	0.08 (0.04 to 0.12)
Low to moderate PTP group (*n *= 258)							
Positive cTnI	77 (54 to 92)	97 (94 to 99)	71 (49 to 87)	98 (95 to 99)	95 (92 to 97)	26.1 (24.0 to 28.1)	0.23 (0.17 to 0.30)
Positive HScTnT	91 (69 to 98)	85 (79 to 89)*	36 (24 to 49)**	99 (96 to 100)	85 (80 to 89)*	6.0 (5.3 to 6.6)	0.11 (0.06 to 0.15)
High PTP group (*n *= 59)							
Positive cTnI	65 (43 to 83)	94 (79 to 99)	88 (62 to 98)	81 (65 to 91)*	83 (71 to 91)**	11.7 (10.1 to 13.4)	0.37 (0.18 to 0.55)
Positive HScTnT	96 (76 to 100)***	67 (49 to 81)***	65 (47 to 81)	96 (78 to 100)	78 (65 to 87)	2.9 (2.3 to 3.4)	0.07 (0 to 0.17)

^a^HScTnT, highly sensitive cardiac troponin T; PPV: positive predictive value; NPV: negative predictive value; Acc: diagnostic accuracy; LR: likelihood ratio. Values are expressed as percentages (except for LR) and their 95% confidence intervall. Positive cTnI > 0.14 μg/L in Pitie-Salpetriere and Ccochin, > 0.06 μg/L in Bicetre; positive HScTnT >0.014 μg/mL. *P < 0.05 versus positive cTnI in all patients; **P < 0.05 versus cTnI in low to moderate PTP group; ***P < 0.05 versus cTnI in high PTP group.

### Net reclassification improvement

Table 3 shows patient classification on the basis of using cTnI or HsTnT to diagnose AMI and highlights the shifts between the two classifications.

### Influence of renal function on cTn performances

**Figure 2:**
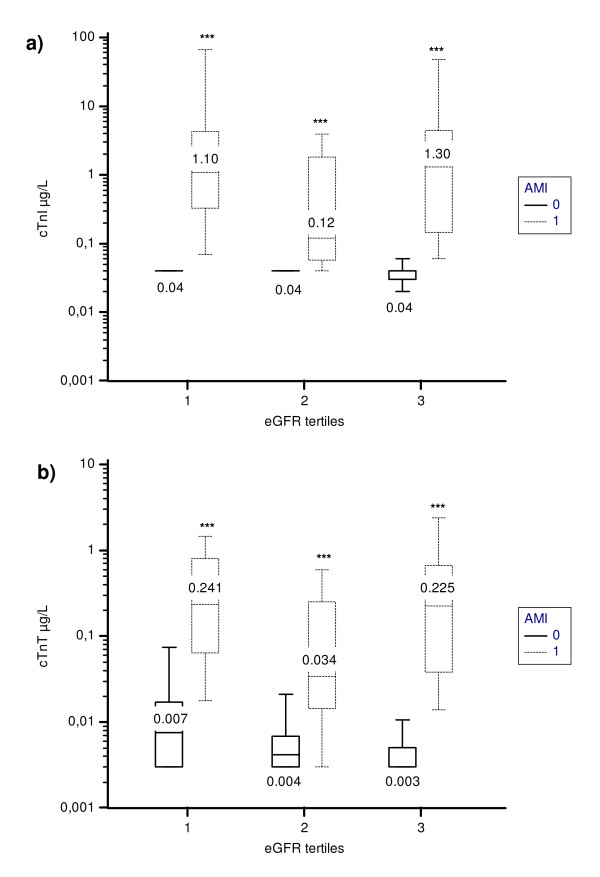
**Boxplots for cTnI (A) and HSTnT (B) values as a function of AMI and according to eGFR tertiles**. ***P < 0.001 versus AMI patients in the same eGFR tertile. Tertile 1 (eGFR < 67.2 mL^-1 ^minute^-1 ^1.73 m^-2^), tertile 2 (eGFR from 67.2 to 86.8 mL^-1 ^minute^-1 ^1.73 m^-2^) and tertile 3 (eGFR ≥ 86.9 mL^-1 ^minute^-1 ^1.73 m^-2^). Medians are indicated for each box.

Patients were classified into tertiles: tertile 1 (estimated glomerular filtration rate (eGFR) < 67.2 ml^-1 ^minute^-1 ^1.73 m^-2^), tertile 2 (eGFR from 67.2 to 86.8 ml^-1 ^minute^-1 ^1.73 m^-2^) and tertile 3 (eGFR ≥ 86.9 ml^-1 ^minute^-1 ^1.73 m^-2^). Cardiac TnI levels were not significantly different across tertiles. However, HsTnT increased significantly across tertiles (*P *< 0.001): the lower the eGFR, the higher the HsTnT value. However, in each eGFR tertile, cTnI and HsTnT levels remained significantly different between AMI and no AMI (*P *< 0.001 for both) (Figure 2). We found no significant differences in the AUCs of cTnI and HsTnT regarding eGFR tertiles, and the optimal threshold value of cTnI did not change across tertiles. Conversely, the optimal threshold value of HsTnT increased only in tertile 1 (0.036 μg/L compared to 0.014 μg/L).

## Discussion

During the past two decades, cTn has been adopted as the preferred biomarker for the diagnosis of acute MI, a position reaffirmed in recent consensus guidelines [14,22]. However, until recently, cTn methods were unable to deliver the requisite analytic performance at the 99th percentile, an extremely low cutoff point within the range of analytic 'noise' in most conventional assays. The present prospective multicenter study of unselected patients who presented to the ED with chest pain of < 6 hours' duration produced major different findings about the new HsTnT assay.

First, the sensitivity of the HsTnT assay remains high at all PTP levels. The excellent sensitivity of 93% was comparable to that found in a previous study (84% to 90% [22]) and significantly higher than conventional cTn (69% in our study and 72% previously described [14]). However, despite its good sensitivity of 91% in the low and moderate PTP groups, the use of HsTnT assays would not allow physicians to rule out AMI in these patients with a unique measurement of HsTnT, as the NPV is not quite perfect, that is, a unique value < 0.014 μg/L cannot avoid a second blood test several hours later to control HsTnT level. It should be noted that in the high PTP group, HsTnT showed excellent diagnostic accuracy, with 93% sensitivity (compared to 80% for cTnI) and 96% NPV (compared to 93% for cTnI). Recently, Januzzi *et al. *[15] showed that HsTnT was able to detect ACS more sensitively than a corresponding conventional cTnT method in a population of low to moderate PTP patients with chest pain.

Second, we confirmed the value of 0.014 μg/L as an optimal threshold [14,22]. We confirmed the high diagnostic accuracy of HsTnT; the AUC of HsTnT was 0.93, similar to that found by investigators in previous studies. Thus, Keller *et al. *[22] and Reichlin *et al. *[14] found AUCs that ranged from 0.94 to 0.96. However, and conversely to other reports, our findings do not show a better AUC for HsTnT than for conventional cTnI measurements. Several reasons could explain this discrepancy.

First, we used cTnI (from Siemens and Beckman Coulter) instead of cTnT as the comparator, thus with a different assay than was previously used, and our comparator cTnI could have slightly better analytical qualities than the one called the 'standard assay' that was used in the Reichlin *et al. *study [14]. Second, in our study, the AUC for cTnI, or 'conventional troponin', that is, the comparator, was 0.94 (95% CI, 0.90 to 0.98), which in fact is included in the 95% CIs of the AUCs of other comparators previously used. For example, Christ *et al. *[23] found an AUC of the standard fourth-generation cTnT assay, that is, its comparator, of 0.89 (95% CI, 0.81 to 0.98). Unfortunately, Keller *et al. *[22] did not detail the 95% CIs of their AUCs for cTn, and Reichlin *et al. *[14] used an old standard assay which in fact underestimated the diagnostic performance of the cTn assay. Other reasons could explain this discrepancy in the AUC of ROC curves for cTnI. Our inclusion criteria differ from those of Reichlin *et al. *[14], Keller *et al. *[22] and others who included patients with chest pain of less than 12 hours' duration with high rates of AMI and unstable angina. Our population markedly differs from those in previous studies. Thus, other conventional cTnT assays (also called third-generation cTnTs, from Roche Diagnostics) that could be used in studies as comparators for HSTnT have been reported to have excellent AUCs. Collinson *et al. *[24] found that at 6 hours postpain, the AUC of cTnT was 0.989 (95% CI, 0.966 to 1.0). However, although the comparison of AUCs remains the most popular metric by which to capture discrimination, it appears that for models containing clinical risk and possessing reasonably good discrimination, very important associations between the biomarker and the end point are required to provide significantly different AUCs. In other words, comparisons of AUCs might be considered powerless in identifying biomarkers of interest in such situations [20]. To address this problem, new ways of evaluating the usefulness of biomarkers have been described, but they are used very rarely in studies evaluating diagnostic tests or biomarkers [14,22]. In the present study, reclassification, for example, NRI, demonstrated that the use of HsTnT with a clinical assessment (including ECG findings) only slightly improved the discriminative power and performance in predicting AMI [14,22,25]. As described in previous studies, we have demonstrated a worsening of specificity and lower PPV of HsTnT measurement compared to those of conventional cTn; that is, we observed an increase in false-positive findings. Last, the present study is the first to investigate the impact of kidney function on HsTnT levels. We found no significant difference in the AUCs of HsTnT regarding eGFR tertiles. Only in tertile 1 was the optimal threshold value of HsTnT increased (0.036 μg/ml compared to 0.014 μg/L).

Conventional cTn is widely used and is recommended for the management of patients presenting with suspected ACS [6]. However, the delay in detecting its elevation prevents early, safe discharge from the ED without repeated negative measurements during the course of 4 to 6 hours. Recent studies have shown excellent diagnostic performance of HsTnT measurement, even with early presentation to the ED [14], and better diagnostic accuracy than cTn [15]. Despite its higher sensitivity, we did not find that HsTnT had better NPV, diagnostic accuracy or AUC, conversely to the findings of previous studies [15]. Furthermore, as expected, specificity and PPV were lower. The clinical setting, time of inclusion, rate of AMI in our patient population and our focus on low or moderate PTP of AMI could explain this discrepancy.

The emergency medicine field would greatly benefit from a new biomarker that eases and hastens the triage of noncardiac chest pain patients. The main incremental value that could have provided a new highly sensitive assay for Tn would have allowed emergency physicians to rule out AMI and discharge patients with a normal Tn value. This study suggests that even when considering only low to moderate PTP patients, the better sensitivity of HsTnT cannot translate into a real clinical improvement. A NPV of 99% can be interpreted as excellent, but this slight gain from that of cTnI is not sufficient to change the conventional method of chest pain investigation in our ED, even in low to moderate PTP patients. This subgroup is the one of most interest in our study, as high PTP patients (and even more so for STEMI patients) are not to be promptly discharged and will more easily undergo further investigations and care.

To rapidly and reliably rule out AMI, the answer may be assessment of a combination of different biomarkers, as suggested by Reichlin *et al. *[26] in their study, where they found that with a copeptin level < 14 pmol/L and a TnT level < 0.01 μg/L, AMI was excluded with 99.7% NPV in an unselected population of chest pain patients.

### Limitations

The main limitation of our study is the small sample of patients, especially patients with AMI. We cannot exclude the possibility that better results might have been found with a larger sample. Our sample is comparable to those used in previous studies, however, and most of all, we believe that the imperfect NPV that we describe herein is the major result of our study, which could not have been corrected by including more patients.

Our study has some other limitations. First, we performed only a single measurement of HsTnT. We did not evaluate its kinetics, which would have been interesting, especially in the 'grey zone' (between 0.014 μg/L and 0.050 μg/L). A second value could have provided more data, as previously described in the Giannitsis *et al. *study [27], which reported that a doubling in the HsTnT concentration within 3 hours of chest pain (with first negative HsTnT and no electrocardiogram abnormality) was associated with a 100% PPV of a diagnosis of NSTEMI.

Second, we used empirical PTP and not a standardised, validated one [17,18]. However, outcomes in the low and moderate PTP population (only nine with confirmed NSTEMI), and differences in clinical characteristics at admission suggested that even though empirical, this evaluation by the clinician was accurate. Furthermore, one of the strengths of our study was that it evaluated differences in diagnostic performance for the HsTnT regarding PTP as demonstrated for D-dimers and empirical suspicion of pulmonary embolism [28]. Another limitation of our study is that different conventional Tn assays have been used at the two study sites with different threshold values and CVs. These assays were used because they were both local and well-understood methods at the time of the study.

Third, we used two different assays for the comparator (that is, conventional TnI): a Siemens cTnI assay in two centres (CCH and PSL) and a Beckman Coulter assay in the third centre (BCT). The ROC curve for the cTnI is, then, a combined ROC curve of two different assays, making it imprecise. However, the two different ROC curves (for each assay) have similar AUCs.

Last, this study was underpowered to find any significant change in the detection of AMI in the low to moderate PTP patients. However, as the NPV is not perfect in our patient population, we expect that this would remain the case with a larger sample.

## Conclusions

We have confirmed that HsTnT is accurate for diagnosis of AMI, with a sensitivity slightly higher than that of conventional cTnI, regardless of PTP of AMI in patients with chest pain presenting to an ED. However, we did not show a better NPV. Intervention studies are clearly warranted to support the use of HsTnT to help ED physicians achieve clinical improvement in treating patients with chest pain and providing them with an early, safe discharge from the hospital.

## Key messages

• Fast and reliable detection of ACS remains a great concern in the ED.

• Novel assays for troponin have been developed and tested recently.

• HsTnT is more sensitive than cTn.

• In this study, the weak gains realised by measuring HsTnT rather than cTn in terms of NPV is not sufficient to change daily clinical practice.

## Abbreviations

ACS: acute coronary syndrome; AMI: acute myocardial infarction; AUC: area under the curve; cTn: conventional troponin; CV: coefficient of variation; ED: emergency department; HsTn: high-sensitivity troponin; LR: likelihood ratio; NPV: negative predictive value; NRI: net reclassification improvement; NSTEMI: non-ST elevation myocardial infarction; PPV: positive predictive value; PTP: pretest probability; ROC: receiver operating characteristic; SD: standard deviation; STEMI: ST elevation myocardial infarction.

## Competing interests

CCG, PR and BR received honoraria from Thermo Fisher Scientific B.R.A.H.M.S. (Hennigsdorf, Germany). PR received an honorarium from bioMérieux, Roche Diagnostics France (Lyon, France).

## Authors' contributions

CCG, BR and PR designed the study. PB, YEC, JCA, BD, FL and CC helped in collecting the data. CC and YF carried out the statistical analyses and the biochemical assays. YF, CCG, BR and PR wrote the paper. All authors read and approved the final manuscript.
